# Effects of combined spinal epidural labor analgesia on episiotomy: a retrospective cohort study

**DOI:** 10.1186/s12871-017-0381-8

**Published:** 2017-06-28

**Authors:** Dandan Zhou, Hui Gong, Shan He, Wei Gao, Qiang Wang

**Affiliations:** 1 Department of Anesthesiology, The Northwest Women’s and Children’s Hospital, Xi’an, Shaanxi Province 710061 China; 2 Department of Anesthesiology, The First Affiliated Hospital of Xi’an Jiaotong University, Xi’an, Shaanxi Province 710061 China

**Keywords:** Combined spinal epidural analgesia, Labor pain, Episiotomy

## Abstract

**Background:**

According to some published studies, neuraxial analgesia may be associated with prolonged labor and an increased risk for instrumental vaginal delivery. However, its effects on episiotomy are unknown. This study aimed to examine the incidence of episiotomy with and without combined spinal–epidural analgesia (CSEA) during labor.

**Methods:**

This was a retrospective cohort study, in which the computerized medical records of nulliparous women with singleton, cephalic and live births were reviewed and women with and without CSEA were matched based on their propensity scores. Univariate and multivariate analyses were used to examine the association between CSEA and the incidence of episiotomy during vaginal delivery.

**Results:**

In the cohort study with 11,994 vaginal deliveries, 5748 received CSEA and 6246 did not receive CSEA. 4116 CSEA women were successfully matched with 4116 Non-CSEA women. In the univariate analysis, the incidence of episiotomy was 47.4% in the CSEA group and 44.7% in the Non-CSEA group. However, after a multivariable logistic regression analysis, CSEA did not increase the risk of episiotomy (adjusted OR, 1.080; 95% confidence interval [CI], 0.988–1.180).

**Conclusions:**

The use of CSEA during labor and vaginal delivery did not increase the risk of episiotomy.

## Background

Painful labor may cause adverse effects for the mother and the fetus. Neuraxial analgesia is the most effective technique for pain relief during labor. In the United States, its use has almost tripled from 22% in 2003 to 61% in 2008 [1], while in France more than 75% women received neuraxial analgesia during labor [2]. However, its effects have been controversial ever since its introduction and have attracted considerable attention and a long-standing interest.

Episiotomy is one of the most common surgical procedures around the world [3]. As an important measure of the quality, National Quality Forum recommended limiting episiotomy because it increased risks of pain, laceration, and anal incontinence [4]. However, few studies have examined the contribution of obstetric risk factors to episiotomy. Neuraxial analgesia is one of the most important external interventions during delivery and according to some previous studies, it may be associated with prolonged labor and an increased rate of instrumental (involving forceps and vacuum extraction) vaginal delivery [5–7]. However, its potential effects on episiotomy are unknown. CSEA is a neuraxial labor analgesic technique that being widely implemented due to its ability to provide rapid and reliable analgesia as well as minimal motor blockade during the first stage of labor. Because of the short history of CSEA, there is insufficient evidence concerning its effects on labor as well as episiotomy and other maternal and neonatal complications. In addition, previous studies have not been able to reach a consensus. Therefore, the main purpose of this study was to assess the association between CSEA and the incidence of episiotomy during vaginal delivery.

## Methods

### Data sources

This was a retrospective cohort study. The reviewed period was from January 1, 2013 to October 31, 2015 in the Northwest Women’s and Children’s Hospital, Xi’an, China. The data were obtained from computerized medical records, which included demographic characteristics, pregnancy information, perinatal outcomes, delivery records and infant characteristics. We used a key word “vaginal delivery” to find out all women giving vaginal delivery from January 1, 2013 to October 31, 2015. Subsequently, two researchers manually extracted patient data from computerized medical records according to the inclusion criteria. The study was approved by the institutional ethics review committee of the Northwest Women’s and Children’s Hospital.

### Study population

The nulliparous women included in this study underwent spontaneous vaginal delivery and had singleton and cephalic presentation. Their gestational periods ranged from 37 weeks to 42 weeks. Multifetal gestations, non-cephalic presentation, intrauterine fetal demise and fetuses involving lethal congenital anomalies were excluded. During the study period, 15,063 women gave birth. Among the excluded women, 125 had multiple gestations, 2162 were multiparas, 98 had non-cephalic presentation, 493 had gestations of less than 37 weeks, 165 underwent labor induction and 26 had missing data in their delivery records. Overall, 11,994 deliveries were included in the present analysis.

### Variables and definitions

The main exposure variable of this study was CSEA during delivery. CSEA was offered to all women upon request, regardless of cervical dilation. Those who refused CSEA were provided with other non-drug analgesic options such as Lamaze, massage, body posture adjustment and exercise. The CSEA anesthesia protocol for women in labor consisted of spinal anesthesia with 2–3 mg of 0.1% ropivacaine and epidural analgesia with a mixture of 0.5 μg.mL^−1^ sulfentanyl plus 1 mg.mL^−1^ ropivacaine, which was delivered in a 6 mL bolus through a lockout interval of 15 min and a baseline infusion rate of 6 mL.h^−1^. Pain intensity was measured using visual analogy score of 0 to 10 points (0 = no pain, 10 = worst pain). Scores of ≤4 were considered to achieve adequate pain relief. If the patients failed to achieve adequate pain relief or if the complete loss of anesthetic levels occurred, the patients underwent epidural re-puncture.

The primary outcome was the incidence of episiotomy. Per the recommendation from the American College of Obstetricians and Gynecologists (ACOG), the clinical indications for episiotomy included imminent and severe perineal rupture, instrumental delivery, shoulder dystocia and a non-reassuring fetal heart rate [8]. The indication for episiotomy was rigorously reviewed by an obstetrician and the operation was performed by a midwife using a routine mediolateral technique. The second outcomes were other maternal outcomes and neonatal complications. Other maternal outcomes included the incidence of instrumental delivery, which was defined as a birth with the use of forceps or vacuum extraction, the incidence of perineal lacerations of above 2 degrees and the incidence of a prolonged second stage of labor. A prolonged second stage of labor was defined as over 3 h of labor for nulliparous women with CSEA and over 2 h of labor for nulliparous women without CSEA. The second stage of labor was from full dilatation of the cervix to the birth of the baby, regardless of whether the fetal presenting part was fully engaged or whether the woman had an urge to push [9]. Postpartum blood loss, defined as the volume of postpartum blood loss recorded 2 h after the delivery, was also treated as a secondary outcome. Neonatal complications were confirmed based on an Apgar score of smaller than 7 at 5 min post delivery and the requirement for neonatal intensive care unit (NICU) admission. Neonatal doctors reviewed neonatal conditions and decided whether the neonates shall be admitted into the NICU.

### Statistical analysis

A propensity score approach was used to adjust for selection bias and observe confounding factors. A propensity score was defined as the probability of receiving CSEA based on individually observed covariates. A 1:1 matching algorithm was used to match CSEA and Non-CSEA women based on gestational age, maternal age and propensity score within a caliper of 0.2 standard deviation of the logit of the propensity score.

Analyses were performed to compare demographic characteristics, pregnancy and delivery complications, and neonatal conditions between these two groups. Baseline clinical data were compared by the *t*-test for continuous variables and the *χ* ^2^ test for categorical variables. A univariate analysis was used to calculate the odds ratio (OR) between CSEA and episiotomy. A multivariate logistic regression in matched cohort was used to adjust maternal age, gestational age, infant birth weight, prolonged second stage of labor and the application of CSEA during delivery. A full logistic regression model was created while subsequent step-wise backward limitation with a *P-*value less than 0.20 as the cutoff point was performed to derive a parsimonious regression model. All covariates entered the model. The instrumental delivery could not be included as a covariate in the logistic regression model because of its collinearity with the prolonged second stage of labor. Results were summarized based on OR or adjusted OR with 95% confidence intervals (95% CI). Statistical analyses were performed using SPSS software, version 21.0 (SPSS, IL, USA). Continuous variables were presented as mean ± standard deviation (SD) while categorical data were presented as numbers and percentages. In all the cases, two-tailed *P-*values less than 0.05 were considered to be statistically significant.

## Results

**Fig. 1:**
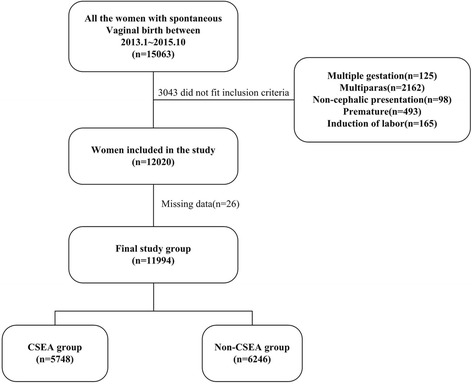
Flow chart detailing the selection of patients included in the retrospective analysis. A total of 15,063 women gave birth in the study period. 11,994 were accorded with the inclusive criteria, and 3069 were excluded. Of these exclusions, 125 were multiple gestations, 2162 were multiparous, 98 were non-cephalic presentation, 493 were premature, 165 were labor induced, and 26 had missing data of labor duration. The final sample comprised 5748 women in the CSEA group and 6246 in the non-CSEA group

In the study with a total of 15,063 women giving birth, 11,994 women met the inclusion criteria while 3069 women were excluded. Among the excluded women, 125 had multiple gestations, 2162 were multiparas, 98 had non-cephalic presentation, 493 had premature gestations, 165 underwent labor induction and 26 had incomplete labor duration data. The reasons for excluding these patients are shown in Fig. 1. The final cohort consisted of 5748 women in the CSEA group and 6246 women in the Non-CSEA group.

The baseline characteristics of the study populations are outlined in Table 1. There were substantial differences in terms of baseline characteristics between the CSEA and the Non-CSEA groups. Patients with an older maternal age or a more advanced gestational age were more likely to choose CSEA. We successfully matched 4116 CSEA patients with 4116 Non-CSEA patients. Baseline characteristics were more comparable between CSEA and Non-CSEA groups in the matched cohort. However, the distributions of body mass index (BMI) and previous pregnancy history were similar in both matched and unmatched groups.

**Table 1:** Maternal demographic and delivery characteristics between the women with and without CSEA. Values are mean (SD) or number (proportion)

	Overall Cohort			Matched Cohort		
Characteristic	Non-CSEA (*n* = 6246)	CSEA (*n* = 5748)	*P-*value	Non-CSEA (*n* = 4116)	CSEA (*n* = 4116)	*P-*value
Maternal age at birth (years)	27.9 ± 2.9	28.0 ± 2.8	0.028	28.0 ± 2.5	28.0 ± 2.8	0.605
BMI^a^(kg.m^−2^)	27.9 ± 5.7	28.0 ± 2.8	0.290	28.0 ± 2.7	27.5 ± 3.1	0.514
Prior miscarriage			0.249			0.796
None	4135 (66.2)	3776 (65.7)		2712 (65.9)	2704 (65.7)	
One	1524 (24.4)	1380 (24.0)		996 (24.2)	988 (24.0)	
Two or more	587 (9.4)	592 (10.3)		399 (9.7)	416 (10.1)	
Gestational age (weeks)	39.6 ± 1.0	39.8 ± 1.0	<0.001	39.7 ± 1.0	39.8 ± 1.0	0.280
Infant birthweight (g)	3288.5 ± 376.4	3372.7 ± 373.9	<0.001	3254.1.4 ± 341.1	3342.5 ± 348.5	<0.001

^a^ *BMI* body mass index

The maternal outcomes and neonatal complications are outlined in Table 2. The use of CSEA was associated with a higher incidence of episiotomy in the matched cohort. Overall, 1838 (44.7%) cases in the Non-CSEA group and 1953 (47.4%) cases in the CSEA group received episiotomy. The rates of instrumental delivery and prolonged second stage of labor were similar in the two groups of the matched cohort. Moreover, no differences were observed in terms of postpartum blood loss and perineal lacerations of above 2 degrees in the two groups.

**Table 2:** Maternal and neonatal outcomes among the women with and without CSEA in Propensity-matched Cohort. Values are mean (SD) or number (proportion)

	Non-CSEA (*n* = 4116)	CSEA (*n* = 4116)	*P-*value
Episiotomy (%)	1838 (44.7)	1953 (47.4)	0.002
Instrumental delivery (%)	74 (2.9)	77 (3.1)	0.434
Prolonged second stage of labor (%)	185 (4.5)	179 (4.3)	0.789
Postpartum blood loss (mL)	188.0 ± 80.1	188.1 ± 80.3	0.781
Perineal lacerations˃2 degrees (%)	111 (2.7)	115 (2.8)	0.840
5-min Apgar score ˂7 (%)	16 (0.4)	8 (0.2)	0.107
NICU admission (%)	12 (0.3)	16 (0.4)	0.458

The incidences of neonatal complications were similar in the two groups of the matched cohort. The percentage of neonates with an Apgar score of smaller than 7 at 5 min post delivery was 0.4% in the Non-CSEA group and 0.2% in the CSEA group. The rate of NICU admission was 0.3% in the Non-CSEA group and 0.4% in the CSEA group.

Within the matched cohort, a total of 3791 women received episiotomy. The incidence of episiotomy was higher in the CSEA group. The univariate analyses showed an increased risk of episiotomy in the CSEA group (OR 1.150, 95% CI 1.055–1.254). However, after adjusting for maternal age, gestational age**,** infant birth weight and prolonged second stage of labor, it was found that CSEA did not increase the risk of episiotomy (adjusted OR 1.080, 95% CI 0.988–1.180) (Table 3). In this model, a prolonged second stage of labor was found to be the main factor for an increase risk of episiotomy (OR 1.765, 95% CI 1.411–2.210). Maternal age at birth (OR 1.081, 95% CI1.064–1.098), gestational age (OR 1.056, 95% CI 1.011–1.103), and infant birth weight (OR 1.001, 95% CI 1.001–1.001) were also found to increase the risk of episiotomy.

**Table 3:** Univariate and multivariate logistical models of predictor risks of episiotomy in Propensity-matched cohort

	With episiotomy *n* (%)	Univariate analysis OR* (95% CI)	Multivariate analysis OR (95% CI)
CSEA		1.150(1.055–1.254)	1.080(0.988–1.180)
Yes	1838 (44.7)		
No	1953 (47.4)		
Maternal age at birth(years)		1.079(1.063–1.096)	1.081(1.064–1.098)
With episiotomy	28.2 ± 2.9		
Without episiotomy	27.6 ± 2.8		
Gestational age (weeks)		1.108(1.063–1.155)	1.056(1.011–1.103)
With episiotomy	39.7 ± 1.0		
Without episiotomy	39.6 ± 1.0		
Prolonged second stage of labor		2.098(1.686–2.609)	1.765(1.411–2.210)
Yes	232 (63.7)		
No	3587 (45.6)		
Infant birthweight (g)		1.001(1.001–1.001)	1.001(1.001–1.001)
With episiotomy	3346.0 ± 356.6		
Without episiotomy	3257.0 ± 334.2		

*OR odds ratio

## Discussion

This study has demonstrated that the use of CSEA during labor and vaginal delivery was not an independent risk factor for episiotomy. Moreover, the incidences of neonatal and maternal complications were similar with and without CSEA using.

Recent studies have shown that episiotomy does not prevent pelvic floor damage and incontinence [10, 11]. On the contrary, it increases the risk of severe perineal tear and wound complications, and impairs sexual function [12, 13]. Consequently, high-risk factors associated with the increased incidence of episiotomy shall trigger more concerns. Although the advantages and safety of using neuraxial analgesia during labor have been well documented, there is continuing controversy over whether neuraxial analgesia causes prolonged labor and increases the rate of operative delivery (cesarean section, forceps and vacuum assisted delivery). Previous studies have shown that local anesthetics interfere with normal expulsive efforts by suppressing the bear-down reflex and hindering the internal rotation of the fetal head, resulting in an increased rate of instrumental delivery and a longer second stage of labor [14–17]. However, it remains unknown whether these adverse effects will lead to an increased rate of episiotomy.

In this retrospective cohort study, we used a propensity score matched analysis to reduce the selection bias in patients receiving CSEA. Although the univariate analysis showed an increased incidence of episiotomy in the CSEA group, the multivariate analysis showed that CSEA was not an independent risk factor. Some previous studies showed that women with a higher maternal age and birth weight were more likely to experience severe pain, prolonged second stage of labor and instrumental delivery. Consequently, these patients may be more likely to receive episiotomy [6]. Consistent with these findings, we found that maternal age, prolonged second stage of labor and a higher birth weight were the main factors increasing the incidence of episiotomy. However, other studies have concluded that neuraxial analgesia is associated with an increased risk of episiotomy and instrumental vaginal delivery. JN Robinson et al. found that epidural analgesia is associated with a more frequent use of operative vaginal delivery and episiotomy [18]. Bodner-Adler B et al. found that women undergoing epidural analgesia are associated with a higher rate of episiotomy [19]. Another study by MG Newman found that epidural analgesia increased the rate of episiotomy [20]. As we know, the effects of CSEA on episiotomy have not been studied before. In our study, a lower concentration of ropivocacine was used for analgesia. Compared with the 0.25% bupivacaine used in the previous studies, 0.1% ropivocacine has a less effect on blocking the motor activity and may have lower influence on labor duration and instrumental delivery. In addition, in two previous studies, episiotomy was not a primary outcome, which means that the confounding factors and patient selection bias in these studies were not well controlled.

In this study, it was also found that the incidence of prolonged second stage of labor was similar in two groups. This finding was inconsistent with some previous study. Gambling and colleagues indicated that the use of CSEA was associated with significantly longer labor compared with the use of intravenous opioids [21]. However, two other studies found that the use of CSEA did not increase the incidence of prolonged labors [22, 23]. There is still insufficient evidence regarding whether or not CSEA prolongs labor, and adequately powered randomized control trials are needed to clarify such questions. Moreover, this study found that the frequency of perineal lacerations of above 2 degrees and postpartum blood loss were similar in the CSEA and Non-CSEA groups. Neonatal complications also showed no significant differences.

This study was novel for several reasons: its major strength was the population-based design with a complete access to the data of labor duration and the maternal and neonatal outcomes. Its large sample size also enabled the authors to evaluate the effects of CSEA based on relatively rare outcomes including prolonged second stage of labor, maternal outcomes and neonatal mobility. Because the study population was analyzed and data on exposure were recorded before the outcome, selection and recall bias was excluded from the study. Furthermore, this study was able to adjust for all possible confounding factors and hence the risk of perinatal outcomes could be comprehensively investigated. The limitations of this study included lack of reporting the pain scores. However, pain intensity was measured using visual analogy score after analgesia, the patients with scores of >4 were treated with supplemental medications or underwent epidural re-puncture in labor. Therefore, we can ensure that all patients in CSEA group received adequacy of the pain relief compared with Non-CSEA group. Another limitation was its historical design. In addition, these data were collected from a single centre and large-scale multicentre clinical trials are still needed.

## Conclusion

In conclusion, it was found that the use of CSEA during labor and vaginal delivery was not an independent risk factor for episiotomy. Future studies are needed to evaluate potential mechanisms and further explain the findings of this study.

## Abbreviations

ACOG: American College of Obstetricians and Gynecologists
BMI: Body mass index.
CI: Confidence intervals
CSEA: Combined spinal epidural analgesia
NICU: Neonatal intensive care unit
OR: Odds ratio
SD: Standard deviation
